# Supplementary material for: network-based constraint to evaluate climate sensitivity
Source: Nat Commun. 2024 Aug 13;15:6942. doi: 10.1038/s41467-024-50813-z (PMC11322302; doi:10.1038/s41467-024-50813-z)
Supplement: Supplementary file 1 — Supplementary Information [file 41467_2024_50813_MOESM1_ESM.pdf]

Supplementary Information for

netCS: a network-based constraint to evaluate Climate Sensitivity

Lucile Ricard[1], Fabrizio Falasca[2], Jakob Runge[3,4,5], Athanasios Nenes[1,6*]

[1]Laboratory of Atmospheric Processes and their Impacts (LAPI), Ecole Polytechnique Fédérale de Lausanne (EPFL), Lausanne, Switzerland

[2]Courant Institute of Mathematical Sciences, New York University, New York, NY USA

[3]German Aerospace Center, Institute of Data Science, 07745 Jena, Germany

[4]Technische Universität Berlin, Berlin, Germany

[5] Center for Scalable Data Analytics and Artificial Intelligence (ScaDS.AI) Dresden/Leipzig, TU Dresden, Germany

[6]Center for the Study of Air Quality and Climate Change (CSTACC), Foundation for Research and Technology Hellas (FORTH), Patras, Greece

*Corresponding author: athanasios.nenes@epfl.ch

This PDF file includes:

Supplementary Notes (1-3)

Supplementary Figures (1-11)

Supplementary Tables (1-6)

Supplementary References

Supplementary Note

Supplementary Note1 - Normality of the distributions

We performed the Shapiro-Wilk test to test the normality distribution of both observable and climate sensitivity values across the ensemble of CMIP6 models. The null hypothesis H_0 states that the data are normally distributed. The p-values of the tests performed with ECS and TCR are both largely superior to significance level α (equals to 0.05). We can not reject H_0 and we conclude both ECS and TCR distributions are normally distributed. When performed with the distance metrics WWD and D_{ACE} , we obtain p-values inferior twice to 0.05. We conclude we can reject the null hypothesis in both cases, and that none of the metrics follow a normal distribution.

Supplementary Note 2 - Perfect model test

We present here the calibration approach to determine the hyperparameter σ_D in the performance weight formula. The approach has been revised since the first one proposed in Sanderson[4] and we apply here the latest perfect model test in date, as seen in ref [5]. The protocol consists in scrolling different σ_D values (between 0.20 and 2.0 that corresponds to a range between 20% and 200% of the value of the median of the distances D_i, with i being the index of the CMIP6 model). For each σ_D, we assume iteratively each model's climate sensitivity as the truth, and we apply the weighting scheme to the remaining models. If 70% of the "true" models have their "true" climate sensitivity that falls within the 10-90 percentile range of the weighted distribution of climate sensitivity, the σ_D is retained as a potential value in a list. Finally, the optimal σ_D value is the minimal value among the list of potential σ_D. We take the minimal value to advantage an aggressive weighting.

In the original protocol proposed in ref[5], the author asked for 80% of models to satisfy the condition to fall in the 10-90 percentile range of the weighted target distribution in order to select a σ_D value. We relax the criterion by asking 19 models out of 27 models to satisfy the condition (~70% of models). The aim is to get weights not too close to the equal weights (equal to $\frac{1}{N}$) used in the unweighted distribution. Finally, the approach allows to get a gradient of weights on both sides of the equal weight value, and to conserve the discriminative information about our models. The weights, like the distance metrics, are spread between 0 and 1, before to be normalized (their sum must be equal to 1).

Supplementary Note- Metrics

For the purpose of constraining climate sensitivity, we reconstructed networks for CMIP6 outputs by fixing the nodes inferred in the reanalysis dataset HadISST. One can also infer nodes in each simulation dataset. However, it is a challenging task to evaluate the reconstructed networks against the reference networks with different number of domains, and a fortiori different number of links. Two network metrics are hereafter presented to evaluate respectively the nodes and the links inferred in a dataset with respect to reference nodes and links. Both metrics offer the advantage of being applicable to networks of varying sizes. *Distance Normalized Mutual Information* (D_{NMI}) scores how well the domains detected in the simulations are close to the true domains. A second metric, *Distance* F_1 (D_{F_1}), scores the presence and absence of links in the simulations with respect to the true links.

Distance Normalized Mutual Information

Distance Normalized Mutual Information (D_{NMI}) scores the domains in term of shapes and sizes, with respect to the "true" domains identified in the reanalysis datasets[1]. D_{NMI} is the complementary measure of the *Normalized Mutual Information* (NMI) metric proposed in ref[2]. The motivation is to evaluate the output of the δ-MAPS algorithm, referred as "domain map", which is a two-dimensional map labeled with 0 ('no domain') and a number between 1 and N, with N being the total number of domains. Each domain receives a different label, but this label is arbitrary and the result is independent of its absolute value. If one grid cell belongs to several domains, the label attributed is the one of its strongest domains.

D_{NMI} comes from the probability and information theory, and evaluates the consistency between the domains inferred in the reanalysis and in the simulations. D_{NMI} takes values between 0 and 1, where D_{NMI} equals to 0 mean

that domain maps are completely correlated and D_{NMI} equals to 1 means that domain maps have no mutual information. We compute D_{NMI} as the complementary value of the normalized mutual info score offered by the scikit learn library on Python [3].

For a domain map D made of d domains and N cells, the probability that a grid cell i falls into the domain d is noted $P(i)$ and is equal to $|d \vee N$. We can then define the entropy $H$of the domain map as:

$$H\,(D) = -\sum^{|\,D\,|} P(i)\log(P(i)) \quad (1)$$

The mutual information between two domain maps D_1 and D_2relies on the notion of joint probability $P(i,j)$:

$$MI\,(D_1, D_2) = \sum^{|D_1|}\sum^{|\,D_2|} P(i,j\,)\,log_2\frac{P(i,j)}{P(i)P(j)} \quad (2)$$

Finally, D_{NMI} between two domain maps D_1 and D_2 is the complementary measure of the normalized mutual information given by the ratio of the mutual information over the averaged entropies of the two domain maps.

$$D_{NMI}(D_1, D_2) = 1 - \frac{MI\,(D_1,D_2)}{mean\,(H\,(D_1),H\,(D_2))} \quad (3)$$

Distance $\mathbf{F_1}$

The causal networks represent characteristic causal fingerprints for each SST dataset, and consist of hundreds of links[1]. In order to evaluate efficiently the detection of causal links in the simulated network with respect to the links revealed in the reference network HadISST, we may calculate the network metric Distance F_1 (D_{F_1}). D_{F_1} scores the presence and absence of links, and tells us how well interconnected are the main modes of temperature variability. It is the complementary measure of network F_1-score, proposed in ref [1], in which the metric was adjusted from the F-score, commonly used to measure classification accuracy, and was applied with success to compare graphs reconstructed from Sea Level Pressure simulations. F_1-score is the harmonic mean of precision (fraction of common links in the network of simulation and the reference network) and recall (fraction of links in the reference network not detected in the network of simulation). The formula of precision (P) and recall (R) are given below, where a True Positive (TP) refers to a link detected both in the reference network and the simulated network, a False Positive (FP) to a link detected in the simulated network only, and a False Negative (FN) to a link detected in the reference network only. The metric is time-independent, which means links detected at different time lags are still counted as TP.

$$P = \frac{TP}{TP+FP} \text{ and } R = \frac{TP}{TP+FN} \quad (4)$$

The two measures R and P are combined to build the F_1 metric, and we define D_{F_1} as its complementary measure, in order to get a 0 value when links are perfectly identical between the two networks:

$$D_{F_1} = 1 - \frac{2.P.R}{P+R} \quad (5)$$

The significance level α and the maximum lag τ_{max} of the PCMCI algorithm modulate the number of links inferred in the graphs, and are set to 0.05 and 3 respectively.

Supplementary Figures

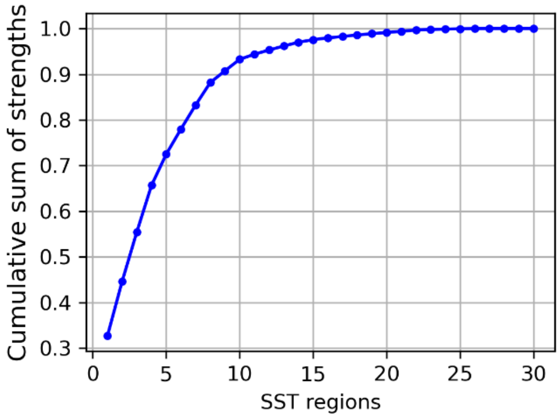

Supplementary Figure 1| Cumulative sum of the proportion of strengths as function of regions.

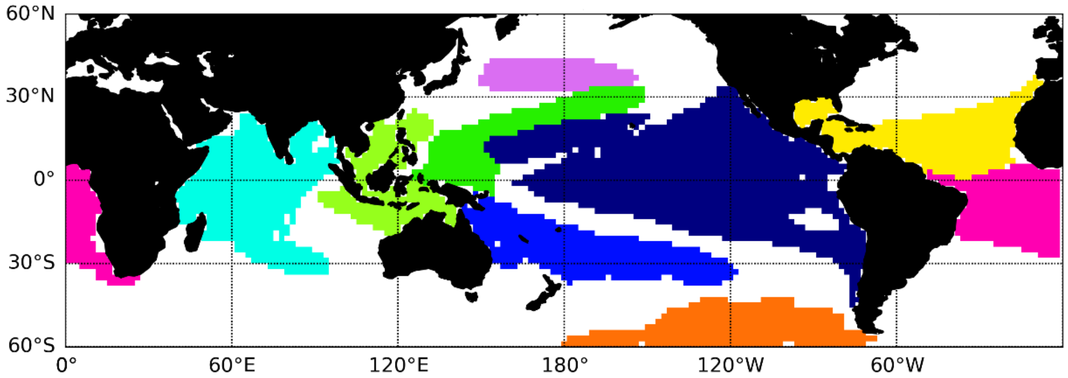

Supplementary Figure 2 | Map of the nine Sea Surface Temperature (SST) regions from which is reconstructed the reference causal network in HadISST dataset, in order to evaluate the teleconnections between the regions. These regions were identified as most climatically-relevant regions, based on their sizes, strengths and geographical locations.

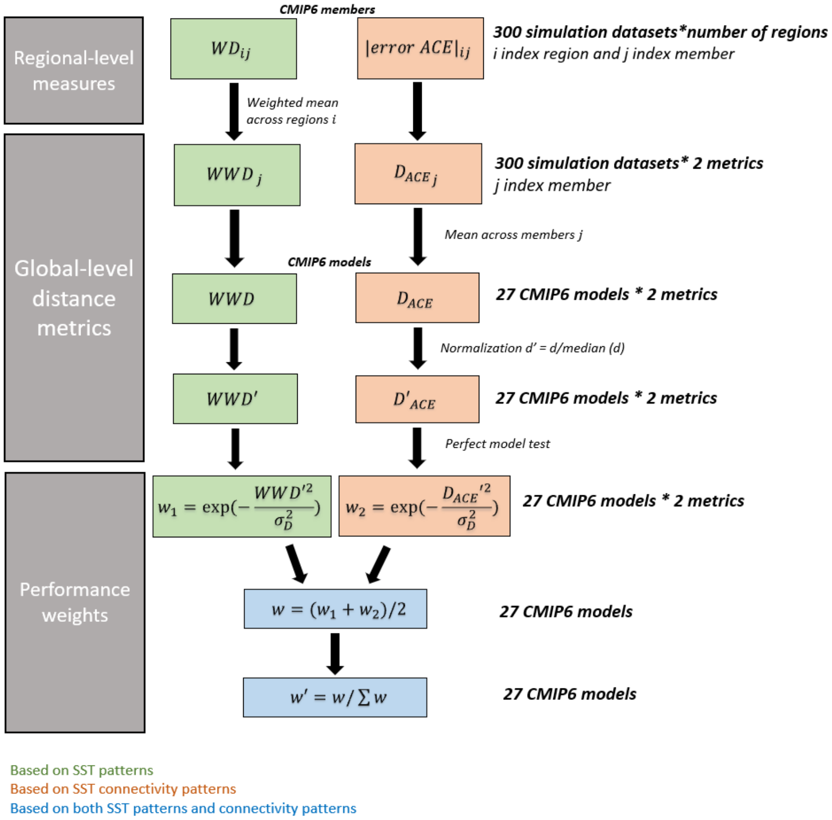

Supplementary Figure 3 | Flowchart. Two regional measures are calculated between model outputs and observations. Global distance metrics are derived from these measures, and averaged across members. Performance weights are derived from normalized metrics, and averaged.

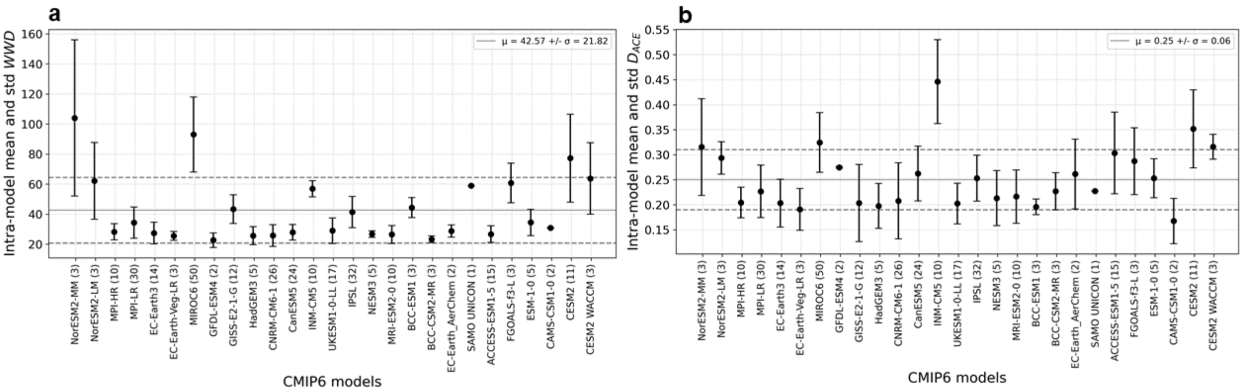

Supplementary Figure 4| Metrics WWD and D_{ACE} in CMIP6 model ensemble. Intra-model mean (dot) and standard deviation (whiskers), and inter-model mean and standard-deviation (solid and dashed lines) for Weighted Wasserstein Distance (WWD) values (a) and Distance Average Causal Effect (D_{ACE}) values (b). The spread of values within models is higher than the spread between models for respectively five and eight CMIP6 models.

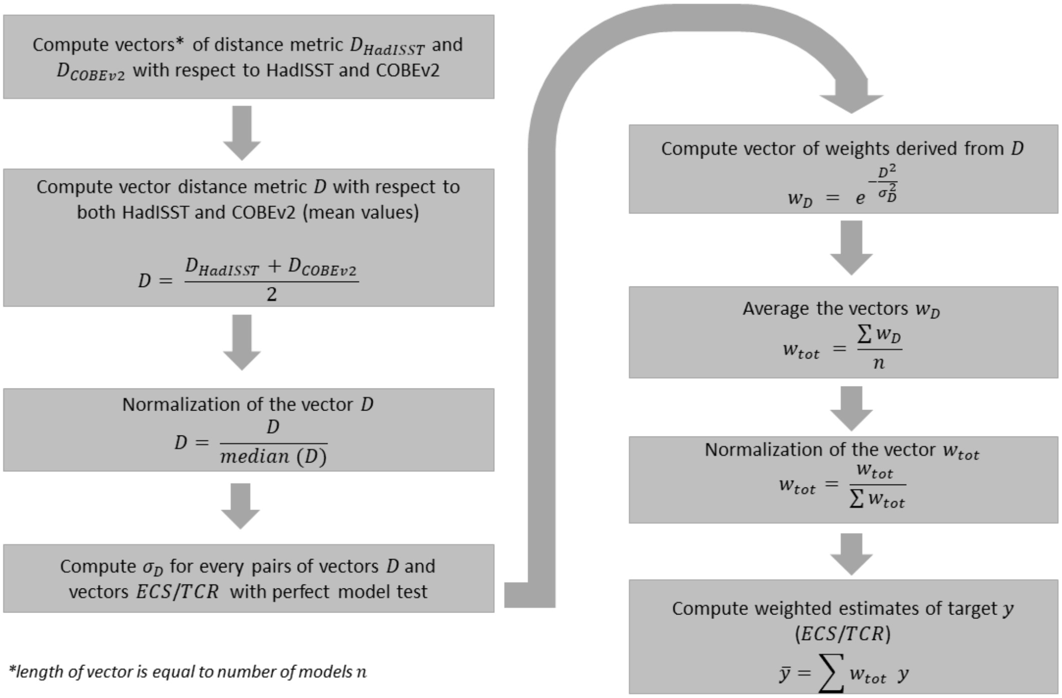

Supplementary Figure 5| Schematic of the weighted estimation of Equilibrium Climate Sensitivity (ECS) and Transient Climate Response (TCR). Estimation of ECS and TCR are based on combination of distance metrics D with respect to two reanalysis datasets (HadISST and COBEv2). In our study, distance D refers to both Weighted Wasserstein Distance (WWD) and Distance Average Causal Effect (D_{ACE}) and the number of distance metrics n is equal to 2. Vector of distance values D is converted to vector of weights w_D through a non-linear transformation, whose intensity is modulated by the factor σ_D. Combination of vector of weights (i.e. mean) is possible and encouraged in order to get a more robust estimate.

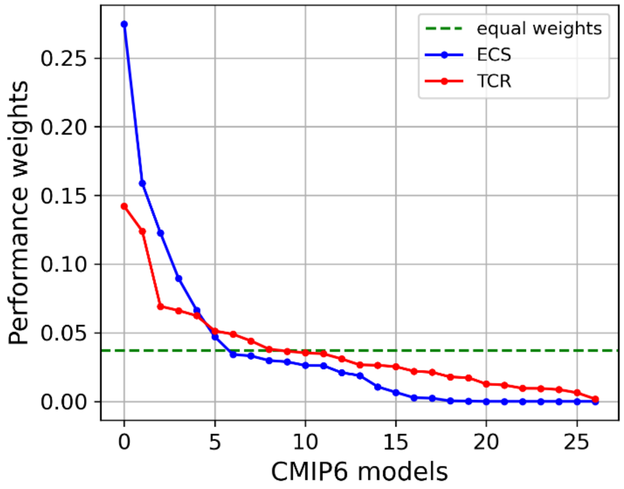

Supplementary Figure 6| Performance weights of Equilibrium Climate Sensitivity (ECS) (blue) and Transient Climate Response (TCR) (red) as function of the CMIP6 models, where models are sorted in decreasing order. Weights are derived from exact same distance metrics values, but the shape parameters determined with the calibration approach lead to a more aggressive weighting for ECS, for which a model contributes to more than 25% of the final estimates. We see 11 models are discarded for ECS estimates, with neglectable weights.

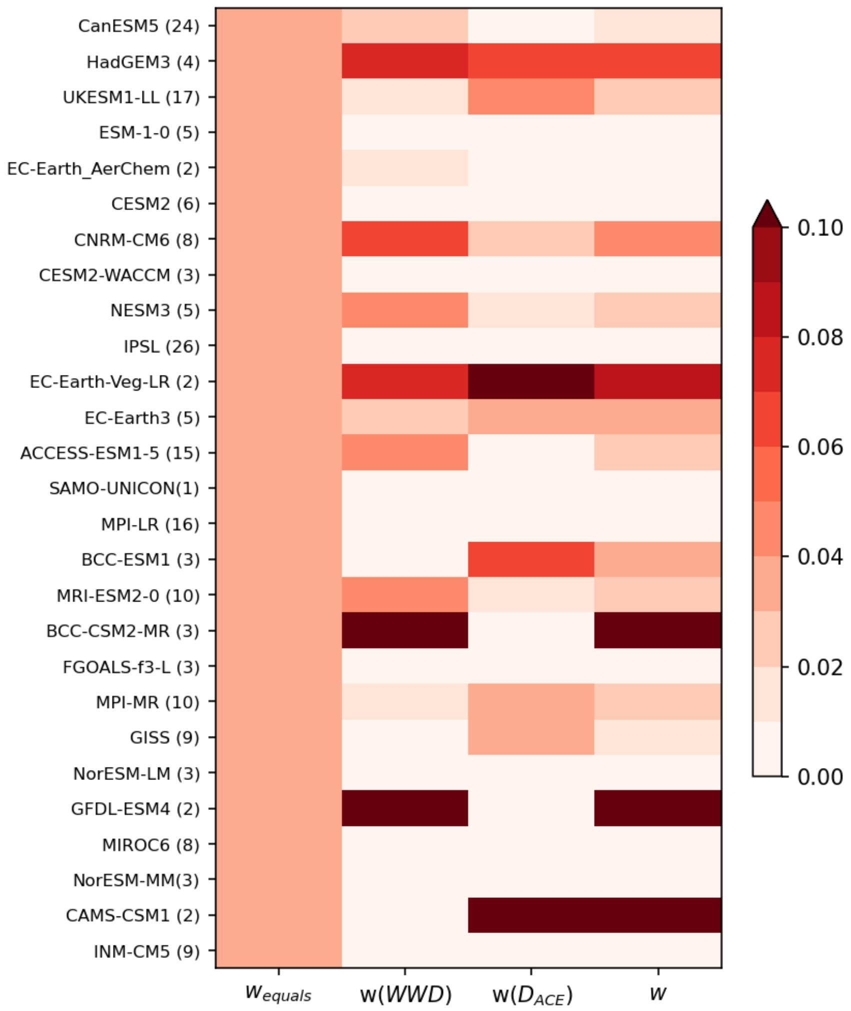

Supplementary Figure 7 | Normalized weights in CMIP6 models. Normalized weights for each CMIP6 models. The left column corresponds to equally weighted models, and the other columns - from left to right - show respectively the weights derived from Weighted Wasserstein Distance (WWD), from Distance Average Causal Effect (D_{ACE}) and the weights averaged. The gradient of red tells us which models are most trustworthy. The darker the red color, the higher the weight. The 27 CMIP6 models are sorted from bottom to top by increasing Equilibrium Climate Sensitivity (ECS). Most important weights are attributed to models at the bottom of the panel, suggesting low ECS models are more realistic.

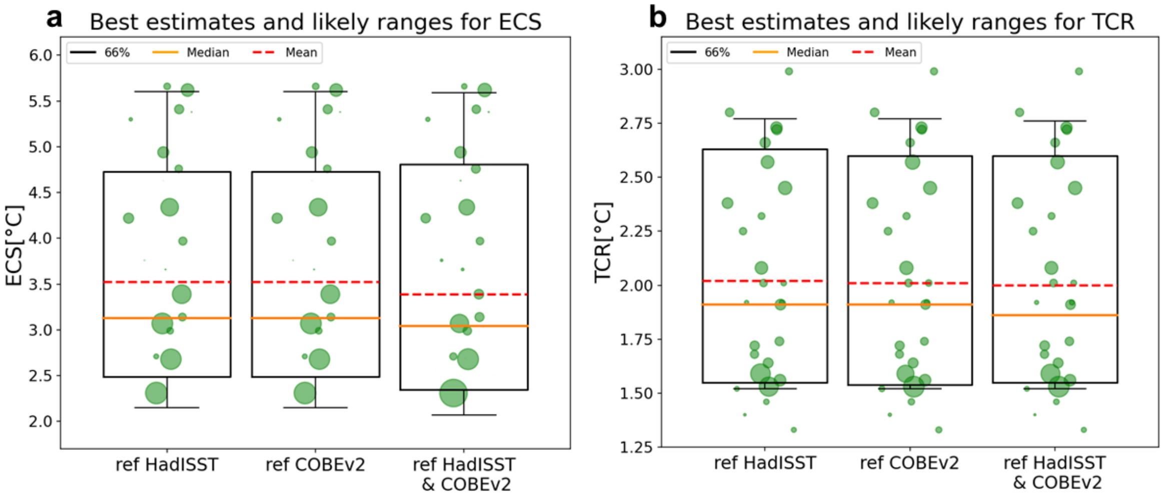

Supplementary Figure 8| Sensitivity of climate sensitivity estimates to reference datasets. Weighted Equilibrium Climate Sensitivity (ECS) estimates (a) and weighted Transient Climate Response (TCR) estimates (b) where weights are derived from distance to HadISST dataset only (left), distance to COBEv2 dataset only (center), and distance to both reference datasets (right). Means (dashed red lines), medians ('central estimate' - orange lines), percentiles 17-83 ('likely range' – boxes) and percentiles 5-95 ('very likely range' – whiskers) are represented.

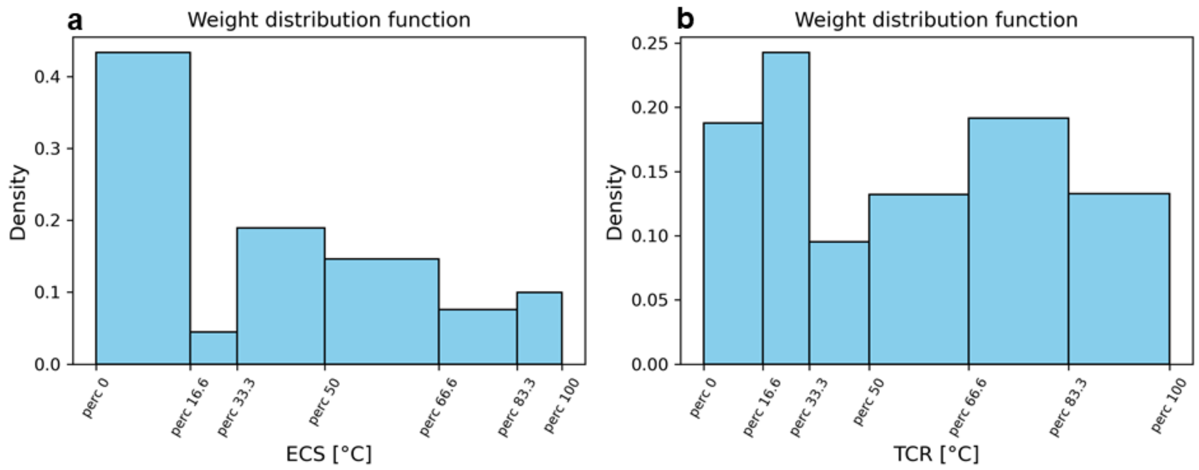

Supplementary Figure 9 | Weight distribution function. Density equals to the sum of the weights of the CMIP6 models comprised in each bin of the Equilibrium Climate Sensitivity (ECS) (a) and Transient Climate Response (TCR) (b) distribution.

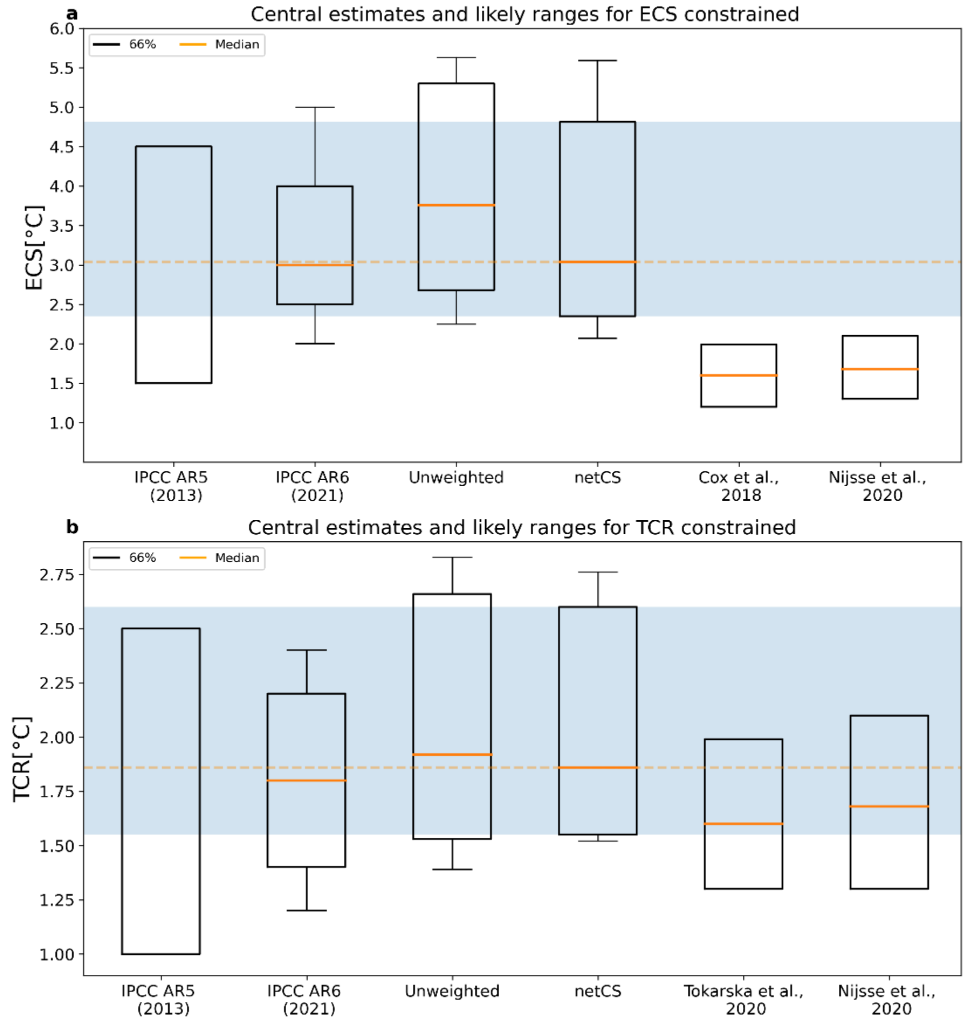

Supplementary Figure 10|Comparison of ECS and TCR estimates with literature. Boxplots of Equilibrium Climate Sensitivity (ECS) (a) and Transient Climate Response (TCR) (b). From left to right : Medians ('central estimate' - orange lines), percentiles 17-83 ('likely range' – boxes) and percentiles 5-95 ('very likely range' – whiskers) extracted from the 2013's IPCC report (based on CMIP5 ensemble)[6] , 2021's IPCC report (based on CMIP6 ensemble)[7], from our initial unweighted CMIP6 distribution, from our final weighted CMIP6 distribution (netCS), and from recent studies based on CMIP6 ensemble[8–10]. The 2021's IPCC estimates are narrower than the 2013's IPCC estimates because based on multiple lines of evidence, but the mean ECS and TCR values and their spread are higher in the CMIP6 ensemble than the CMIP5 ensemble.

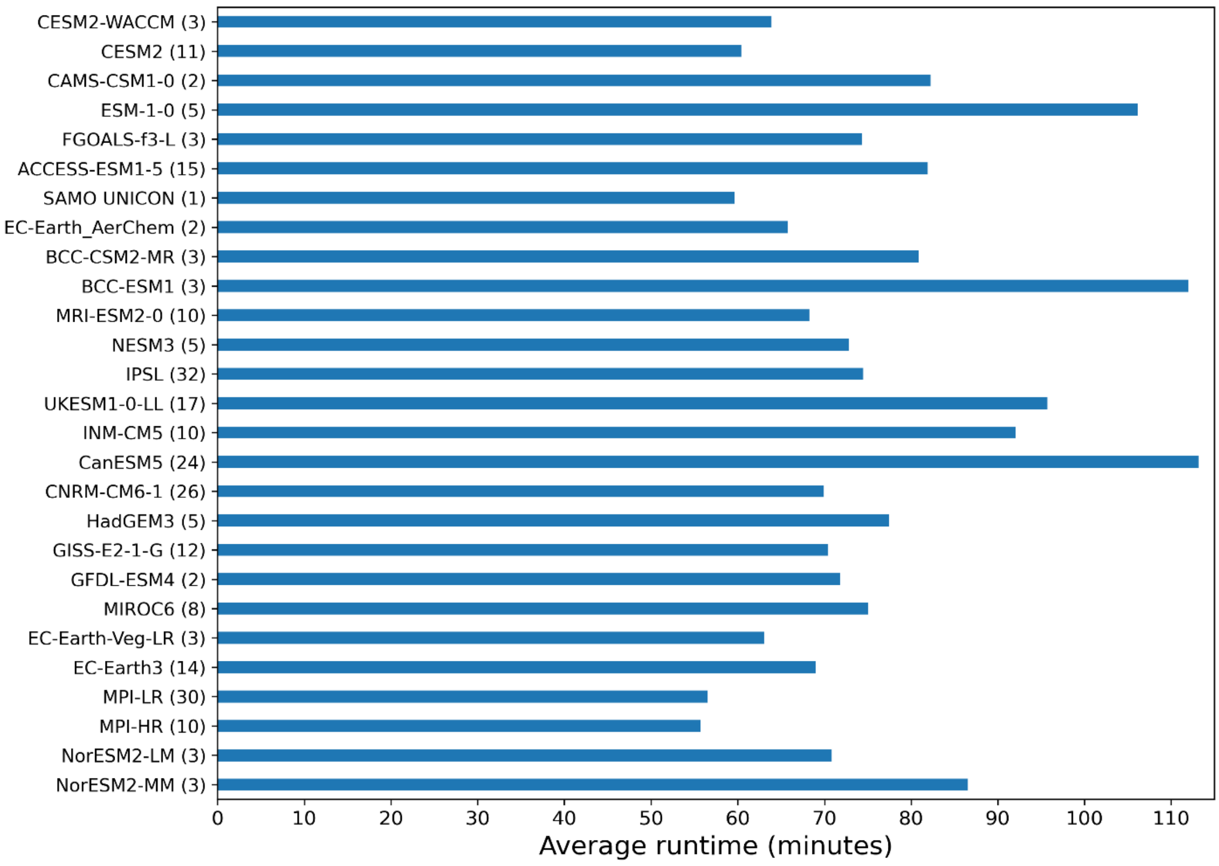

Supplementary Figure 11| Runtime of domain inference with δ-MAPS. Average time of execution of the domain inference step of δ-MAPS algorithm, for the different CMIP6 models. The time is averaged over all the different simulations of each model. Hyperparameters k and α are set to 8 and 0.01 respectively. The operation takes between 55 minutes and 2 hours depending on the models. Depending on the number of domains detected in the initialization, the algorithm may be more or less long to run during the merging and expansion step.

Supplementary Tables

Model (runs)	ECS (°C)	TCR (°C)	D_{NMI}	WWD	D_{F_1}	D_{ACE}
NorESM2-MM (3)	2.50	1.33	0.423	104.03	0.502	0.315
NorESM2-LM (3)	2.69	1.46	0.431	62.16	0.522	0.294
MPI-ESM1-2-HR (10)	2.99	1.64	0.440	28.22	0.523	0.204
MPI-ESM1-2-LR (30)	3.66	2.01	0.431	34.36	0.520	0.227
EC-Earth3 (14)	4.22	2.38	0.371	27.4	0.542	0.203
EC-Earth AerChem (2)	5.30	2.8	0.387	25.53	0.539	0.191
EC-Earth3-Veg-LR (3)	4.34	2.57	0.382	93.04	0.548	0.324
MIROC6 (50)	2.56	1.52	0.405	22.67	0.522	0.275
GFDL-ESM4 (2)	2.68	1.53	0.376	43.36	0.545	0.203
GISS-E2-1-G (12)	2.71	1.68	0.421	25.65	0.529	0.198
HadGEM3-GC31-LL (5)	5.62	2.45	0.398	25.77	0.527	0.208
CNRM-CM6-1 (26)	4.94	2.08	0.402	27.89	0.544	0.262
CanESM5 (24)	5.66	2.66	0.425	56.90	0.540	0.446
INM-CM5 (10)	1.93	1.40	0.437	28.96	0.551	0.202
UKESM1-0-LL (17)	5.41	2.72	0.405	41.37	0.521	0.253
IPSL-CM6A-LR (32)	4.63	2.32	0.393	26.71	0.541	0.213
NESM3 (5)	4.76	2.73	0.383	26.46	0.530	0.216
MRI-ESM2-0 (10)	3.14	1.56	0.406	44.34	0.542	0.196
BCC-ESM1 (3)	3.39	1.74	0.429	23.26	0.558	0.227
BCC-CSM2-MR (3)	3.07	1.59	0.416	28.72	0.561	0.261
SAMO-UNICON (1)	3.76	2.25	0.396	58.85	0.522	0.228
ACCESS-ESM1-5 (15)	3.97	1.91	0.423	26.67	0.535	0.304
E3SM-1-0 (5)	5.38	2.99	0.406	60.75	0.514	0.287
FGOALS-f3-L (3)	3.03	2.01	0.396	34.45	0.552	0.253
CAMS-CSM1-0 (2)	2.31	1.72	0.41	30.84	0.566	0.168
CESM2 (6)	5.30	2.04	0.383	77.25	0.528	0.352
CESM2 WACCM (3)	4.90	1.92	0.400	63.75	0.526	0.316
**Mean μ**	3.88	2.03	0.406	42.57	0.535	0.253
**Standard Deviation σ**	1.15	0.48	0.019	21.82	0.015	0.061

Supplementary Table 1|Network metric values of CMIP6 models. List of Equilibrium Climate Sensitivity (ECS) and Transient Climate Response (TCR) values [9], and ensemble mean metric values for each of the 27 CMIP6 models. Red color indicates the maximum ECS and TCR value (the warmest models), and the maximum metric value (the farthest models to the ground truth). Green color indicates the minimum ECS and TCR values (the coldest model) and the minimum metric value (the closest models to the ground truth). The mean and variability of each column are also indicated.

α-level	Number of past cross-links discovered in HadISST	Proportion of past cross-links discovered
0.01	16	7%
0.05	34	16%
0.20	79	37%

Supplementary Table 2 | Number of past cross-links detected with PCMCI in the HadISST dataset. For different α-levels, corresponding number of cross-links and proportion of cross-links detected. For a network made of n nodes, there are $\binom{n}{2}$ potential undirected links. For our network made of nine nodes, with a maximum time lag equals to three months, there are potentially 216 past cross-links which may be revealed in the causal structure. The influence of the hyperparameter α (significance level) is important, especially since very few links are detected even when α is equal to 0.20, which shows how strict the link detection is. The lower the significance level, the stricter.

σ_D	ECS(°C)	TCR(°C)
$w(WWD)$	0.32	0.34
$w(D_{ACE})$	0.31	1.07

Supplementary Table 3| σ_D values determined with the perfect model test. The different σ_D values for weights derived from vectors of Weighted Wasserstein Distance (WWD) and Distance Average Causal Effect (D_{ACE}) values for the different climate sensitivity metrics Equilibrium Climate Sensitivity (ECS) and Transient Climate Response (TCR). The lower σ_D, the more aggressive the weighting.

σ	ECS (°C)	TCR (°C)
$w(WWD)$	0.073	0.067
$w(D_{ACE})$	0.103	0.015
$w\ (WWD, D_{ACE})$	0.061	0.035

Supplementary Table 4 | Variability of weights. Standard deviation of the weights derived from Weighted Wasserstein Distance (WWD)and Distance Average Causal Effect (D_{ACE}) and for the different climate sensitivity metrics Equilibrium Climate Sensitivity (ECS) and Transient Climate Response. The variability across the weights of the models is more important with ECS as target metric.

Approach	Central estimate (°C)	Percentile 17-83 (length)	Percentile 5-95 (length)
IPCC AR6 (2021)	3.00	2.5 to 4.0 (1.5)	
Unweighted	3.76	2.68 to 5.3 (2.62)	2.25 to 5.63 (3.38)
netCS	3.04	2.35 to 4.81 (2.46)	2.07 to 5.59 (3.52)

Supplementary Table 5 | Equilibrium Climate Sensitivity estimates derived from netCS approach. Median ('central estimate') and percentiles 17-83 ('likely range') extracted from the 2021's IPCC report (first column), from our initial unweighted CMIP6 distribution (second column) and from our final weighted CMIP6 distribution "netCS" (third column). Final estimates can be rigorously compared with the unweighted ones since assessed ranges of IPCC are based on multiple lines of evidences.

Approach	Central estimate (°C)	Percentile 17-83 (length)	Percentile 5-95 (length)
IPCC AR6 (2021)	1.80	1.4 to 2.2 (0.8)	
Unweighted	1.92	1.53 to 2.66 (1.13)	1.39 to 2.83 (1.44)
netCS	1.86	1.55 to 2.60 (1.05)	1.52 to 2.76 (1.24)

Supplementary Table 6 | Transient Climate Responses estimates derived from netCS approach. Median ('central estimate') and percentiles 17-83 ('likely range') extracted from the 2021's IPCC report (first column), from our initial unweighted CMIP6 distribution (second column) and from our final weighted CMIP6 distribution "netCS" (third column). Final estimates can be rigorously compared with the unweighted ones since assessed ranges of IPCC are based on multiple lines of evidences.

Supplementary References

1. Nowack, P., Runge, J., Eyring, V. & Haigh, J. D. Causal networks for climate model evaluation and constrained projections. *Nat. Commun.* **11**, 1415 (2020).
2. Falasca, F., Bracco, A., Nenes, A. & Fountalis, I. Dimensionality Reduction and Network Inference for Climate Data Using δ-MAPS: Application to the CESM Large Ensemble Sea Surface Temperature. *J. Adv. Model. Earth Syst.* **11**, 1479–1515 (2019).
3. Pedregosa, F. *et al.* Scikit-learn: Machine Learning in Python. *Mach. Learn. PYTHON*.
4. Sanderson, B. M., Wehner, M. & Knutti, R. Skill and independence weighting for multi-model assessments. *Geosci. Model Dev.* **10**, 2379–2395 (2017).
5. Brunner, L. *et al.* Reduced global warming from CMIP6 projections when weighting models by performance and independence. *Earth Syst. Dyn.* **11**, 995–1012 (2020).
6. Pachauri, R. K. *et al. Climate Change 2014: Synthesis Report. Contribution of Working Groups I, II and III to the Fifth Assessment Report of the Intergovernmental Panel on Climate Change*. *EPIC3Geneva, Switzerland, IPCC, 151 p., pp. 151, ISBN: 978-92-9169-143-2* 151 (IPCC, 2014).
7. Forster, P. *et al.* The Earth's energy budget, climate feedbacks, and climate sensitivity. (2021).
8. Cox, P. M., Huntingford, C. & Williamson, M. S. Emergent constraint on equilibrium climate sensitivity from global temperature variability. *Nature* **553**, 319–322 (2018).
9. Nijsse, F. J. M. M., Cox, P. M. & Williamson, M. S. Emergent constraints on transient climate response (TCR) and equilibrium climate sensitivity (ECS) from historical warming in CMIP5 and CMIP6 models. *Earth Syst. Dyn.* **11**, 737–750 (2020).
10. Tokarska, K. B. *et al.* Past warming trend constrains future warming in CMIP6 models. *Sci. Adv.* **6**, eaaz9549 (2020).
